# hsa-miR-376c-3p Regulates Gastric Tumor Growth Both* In Vitro* and* In Vivo*

**DOI:** 10.1155/2016/9604257

**Published:** 2016-11-14

**Authors:** Lin Tu, Enhao Zhao, Wenyi Zhao, Zizhen Zhang, Defeng Tang, Yeqian Zhang, Chaojie Wang, Chun Zhuang, Hui Cao

**Affiliations:** Department of Gastrointestinal Surgery, Ren Ji Hospital, School of Medicine, Shanghai Jiao Tong University, Shanghai 200127, China

## Abstract

*Background*. In recent studies, aberrant expression of various microRNAs (miRNAs) is reported to be associated with gastric cancer metastasis.* Method*. Overexpression construct and inhibitor of hsa-miR-376c-3p were expressed in human gastric adenocarcinoma cell line SGC-7901. The expression level of tumor related genes was detected by qPCR, western blot, and immunostaining. Cell apoptosis was determined by flow cytometry. Xenograft of SGC-7901 cells was used to elucidate the function of hsa-miR-376c-3p in gastric tumor growth* in vivo*.* Result*. Expression of hsa-miR-376c-3p was detected in SGC-7901 cells. Downregulation of hsa-miR-376c-3p increased the expression level of BCL-2 and decreased the expression of smad4 and BAD. On the contrary, overexpression of hsa-miR-376c-3p increased the expression of BAD and smad4, while it led to the decreasing expression level of BCL-2. Overexpression of hsa-miR-376c-3p also promoted cell apoptosis* in vitro* and inhibited gastric tumor growth* in vivo*. Furthermore, the expression of BCL-2 was higher and expression of smad4 and BAD was lower in tumor tissue than the tissue adjacent to tumor from gastric cancer patients.* Conclusion*. This study demonstrated that hsa-miR-376c-3p plays an important role in the inhibition of gastric tumor growth and tumor related gene expression both* in vitro* and* in vivo*.

## 1. Introduction

Gastric cancer (also known as stomach cancer) is a significant health problem. Globally gastric cancer is the fifth most common malignancy worldwide and the third leading cause of death from cancer across the two genders. Gastric cancer accounts for approximately one million new cases and more than 700,000 cancer-related deaths annually in the world [1–3]. Although several types of treatments have been developed for gastric cancer in clinical trials, drug resistance is still the major issue causing the failure in gastric cancer treatment. Besides the drug resistance, the other vital cause of cancer-related death in gastric cancer patients is metastasis, which involves a series of complex mechanisms [4]. More than half of gastric cancer patients will die within six months due to regional recurrence or distant metastasis [5]. Many signaling molecules such as TGF-*β*, matrix metalloproteinase- (MMP-) 2, MMP-9, *β*-catenin, and vascular endothelial growth factor (VEGF) have been shown to affect tumor metastasis [6]. Previous studies indicated that the dysregulation and dysfunction of these signaling pathways leads to mutations in critical genes responsible for the proliferation, apoptosis, and metastasis of malignant gastric tumor cells [7].

Recent studies indicate that abnormal expression of microRNAs (miRNAs) is an important factor for tumor metastasis [8]. miRNA is a small noncoding RNA molecule (containing about 22 nucleotides) found in plants, animals, and some viruses and played critical roles in cancer [9]. Recent studies have identified altered and aberrant regulation of miRNAs in gastric cancer, mediating the tumor growth, metastasis, and invasion [10–15]. Thus, there is a great clinical interest to further investigate the molecular mechanisms of miRNAs aiding in the development of novel strategies for gastric cancer prevention, diagnosis, and treatment in order to improve the clinical outcome of gastric cancer patients.

In this study, we investigated the role of hsa-miR-376c-3p in human gastric adenocarcinoma cell line SGC-7901. We found that regulation of hsa-miR-376c-3p played role in the tumor cell growth both* in vitro* and* in vivo*. This provided an indication to support the potential clinical application. In the future, the development of hsa-miR-376c-3p as a novel therapeutic target for the treatment of gastric cancer may be applicable.

## 2. Methods and Materials

### 2.1. Cell Culture

Human gastric adenocarcinoma cell line SGC-7901 was purchased from Cancer Research Institute of Beijing (China). Cells were cultured in RPMI 1640 medium, supplemented with 10% (v/v) fetal bovine serum, 100 mg/mL penicillin, and streptomycin (all purchased from Life Technologies) at 37°C with 5% CO_2_.

### 2.2. Constructs of hsa-miR-376c-3p Inhibitor and Overexpression Plasmid

Inhibitor negative control and hsa-miR-376c-3p inhibitor miRNA were purchased from ABM (Canada). Sequence of hsa-miR-376c-3p was cloned into PCI vector. Cell transfection was conducted by Lipofectamine 2000 (Life Technologies). All the protocol for cell transfection was strictly followed by the product manual.

### 2.3. Analysis of Cell Cycle Phase by Flow Cytometry

Forty-eight hours following transfection, SGC-7901 cells were resuspended in PBS twice before fixation by adding dropwise to 95% precooled ethanol. Prior to analysis, the cells were warmed, centrifuged at 450 g for 5 min, and resuspended twice in PBS and then stained with PI (containing RNase A at 50 *μ*g/mL) at room temperature in the dark for 30 min. The DNA content was analyzed by flow cytometry using the CellQuest program (Becton-Dickinson and Co., USA).

### 2.4. Western Blotting

2 *μ*g cell lysates were loaded on each lane of 10% polyacrylamide gel and then blotted onto a polyvinylidene difluoride (PVDF) membrane. After blocking with a PBST containing 5% nonfat dry milk, the membrane was incubated with antibodies against BCL-2, BAD, smad4, and GAPDH (Cell Signaling Technologies, USA). Peroxidase-linked anti-rabbit IgG (Life Technologies) were used as secondary antibodies. These proteins were visualized by using an ECL western blotting detection kit (Amersham Biosciences).

### 2.5. Xenograft Tumor Models

According to previous study [16], inoculation area of the mice was cleaned and sterilized with ethanol and iodine solutions. SGC-7901 cells (2 × 10^6^ cells in 200 *μ*L PBS) transfected with different constructs were injected subcutaneously into right armpit of 6-week-old BALB/c nude mice. Obvious tumor was observed 4 weeks after cell injection. The implanted mice were observed daily until 60 days. Tumor volume (*V*) was calculated using the following equation: *V* = (*a* ^2^ × *b*)/2, where *a* is the width of the tumor (small diameter) and *b* is the length (large diameter) (mm).

### 2.6. Immunohistochemistry

The tumor sections from xenograft tissues were washed in PBS, blocked for 60 min in 0.3% Triton X-100 in phosphate-buffered saline with 5% bovine serum albumin, and then incubated at 4°C with anti-BAD, anti-BCL-2, and anti-smad4 antibodies (purchased from Cell Signaling Technology) overnight. HRP-conjugated secondary antibody was applied to the slides and incubated for 1 hour at room temperature. Lastly, DAB/H_2_O_2_ was added to the surface of the slide to develop the color. The slides were visualized by using a Nikon ECLIPSE 90i.

### 2.7. RNA Extraction and qRT-PCR

Total RNA extraction was performed using TRIzol reagent (Life Technologies) according to the manufacturer's instruction. Two micrograms of total RNA extracted from the cells was subjected to reverse transcription (RT). Synthesis of cDNA was performed by using one-step RT-PCR kit from Takara. SYBR Green (Toyobo) RT-PCR amplification and real time fluorescence detection were performed using ABI 7300 real time PCR thermal cycle instrument (ABI, USA), according to the supplied protocol. Relative gene expression was calculated by the ΔΔCt method.

### 2.8. Statistical Analysis

Raw data were analyzed using Microsoft Excel software. All data were presented as mean ± standard deviation. The difference among all the groups was determined by paired *t*-test. A *p* value less than 0.05 is considered as significantly different and less than 0.01 is considered as very significantly different, while a *p* value more than 0.05 is considered as not significantly different.

## 3. Results

### 3.1. hsa-miR-376c-3p Regulated SGC-7901 Cell Apoptosis

**Figure 1:**
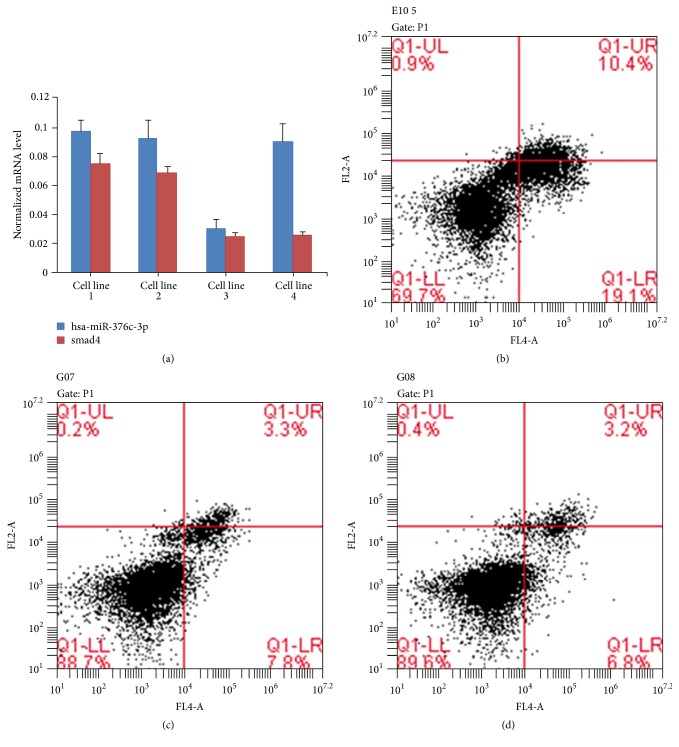
(a) Real time PCR results indicated the expression of hsa-miR-376c-3p and smad4 in four different gastric cancer cell lines. Measurement of apoptotic cell ratio was conducted by flow cytometry in hsa-miR-376c-3p overexpressed (b), nontransfected (c), and empty vector-transfected (d) SGC-7901 cells.

**Figure 2:**
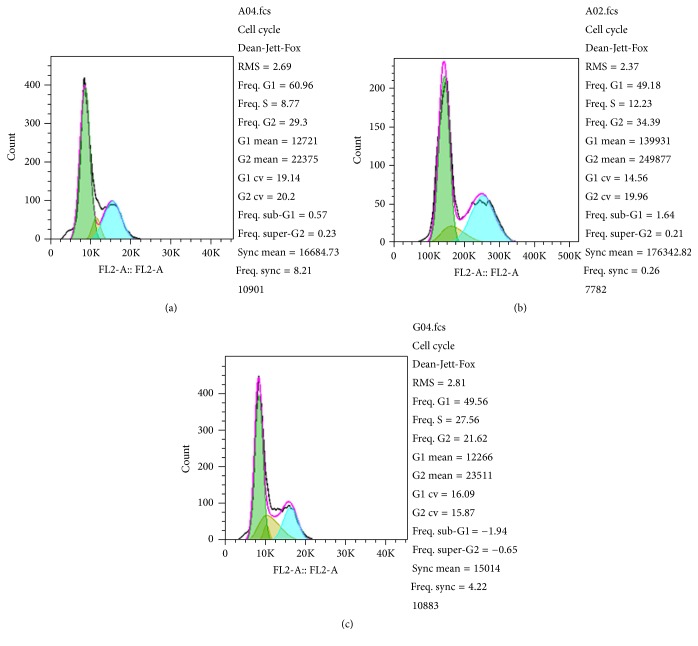
Cell cycle analysis was performed by flow cytometry in hsa-miR-376c-3p overexpressed (a), nontransfected (b), and empty vector-transfected (c) SGC-7901 cells.

To elucidate the role of hsa-miR-376c-3p in gastric tumor growth, we firstly determined the expression level of hsa-miR-376c-3p and smad4 in four cell lines. The quantitative reverse transcription PCR result indicated that both hsa-miR-376c-3p and smad4 were expressed in the gastric cancer cell line (Figure 1(a)). Since hsa-miR-376c-3p was expressed in the gastric adenocarcinoma cell line SGC-7901, we determined whether the regulation of hsa-miR-376c-3p affected the cell viability. The hsa-miR-376c-3p overexpression group exhibited the enhanced cell apoptosis (Figure 1(b)) and increases the cell ratio at G1 phase (Figure 2(a)) compared to the nontransfected group (Figures 1(c) and 2(b)) and empty vector group (Figures 1(d) and 2(c)); however, there is no significant difference.

BAD/BCL-2 signaling was involved in hsa-miR-376c-3p-mediated apoptosis

**Figure 3:**
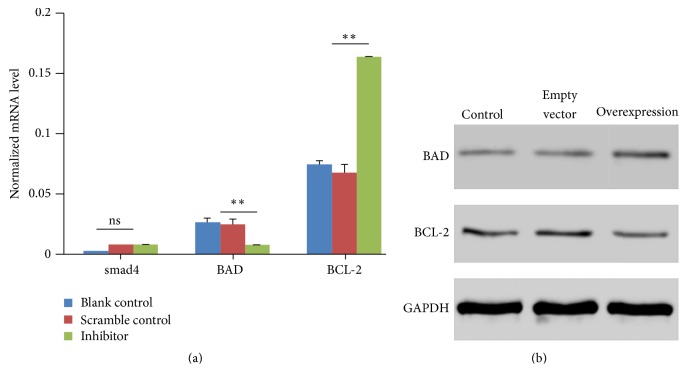
The expression alteration of BAD and BCL-2 was determined by real time PCR (a) and western blotting (b) in hsa-miR-376c-3p inhibitor transfected, nontransfected, and scramble control-transfected SGC-7901 cells. The significance was calculated by paired *t*-test. “ns” represented not significant. ^*∗∗*^ *p* < 0.01.

Furthermore, we found the regulation of hsa-miR-376c-3p could alter the expression level of some cancer-related genes. As shown in Figure 3(a), the miRNA inhibition drastically decreased the mRNA level of apoptotic gene BAD and increased the antiapoptotic gene BCL-2. However, no significant expression alteration in smad4 was observed compared to scramble control group. Besides, the miRNA overexpression significantly increased the protein expression level of apoptotic gene BAD and decreased the antiapoptotic gene BCL-2 (Figure 3(b)). Collectively, these results indicated that hsa-miR-376c-3p expression regulated the gastric tumor cell growth* in vitro* through BAD/BCL-2 pathway.

### 3.2. Overexpression of hsa-miR-376c-3p Inhibited the Gastric Tumor Growth* In Vivo*

**Figure 4:**
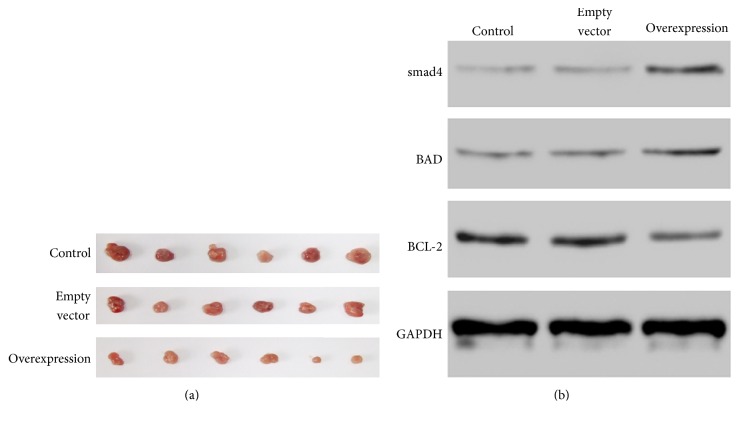
(a) Tumor samples from xenograft of wide type, empty vector-transfected, and hsa-miR-376c-3p overexpressed SGC-7901 cells. (b) Western blotting showed the expression change of smad4, BAD, and BCL-2 among three different groups.

**Figure 5:**
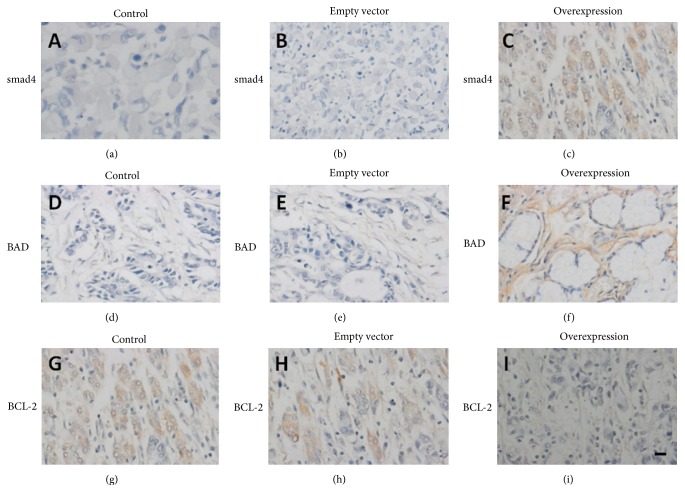
Immunostaining analysis indicated expression alteration in smad4 (a–c), BAD (d–f), and BCL-2 (g–i) in tumor sections from xenograft of wide type, empty vector-transfected, and hsa-miR-376c-3p overexpressed SGC-7901 cells.

Next, we detected the effect of hsa-miR-376c-3p on gastric tumor growth* in vivo*. Xenograft of SGC-7901 cells leads to the tumor information in the BALB/c nude mice (Figure 4(a)). Xenograft of SGC-7901 cells transfected with empty vector did not change the tumor size, while the overexpression of hsa-miR-376c-3p negatively affected the* in vivo* tumor growth compared to the controls (Figure 4(a)). Besides, the western blotting data showed that overexpression of hsa-miR-376c-3p increased the expression of BAD and smad4 and decreased the antiapoptotic gene BCL-2 (Figure 4(b)). However, the transfection of empty vector did not show any significant change in those tumor related proteins (Figure 4(b)). Besides, immunostaining result further confirmed expression alteration in smad4, BAD, and BCL-2 when hsa-miR-376c-3p was overexpressed (Figure 5).

### 3.3. Expression of hsa-miR-376c-3p in Tumor Samples from Gastric Cancer Patients

**Figure 6:**
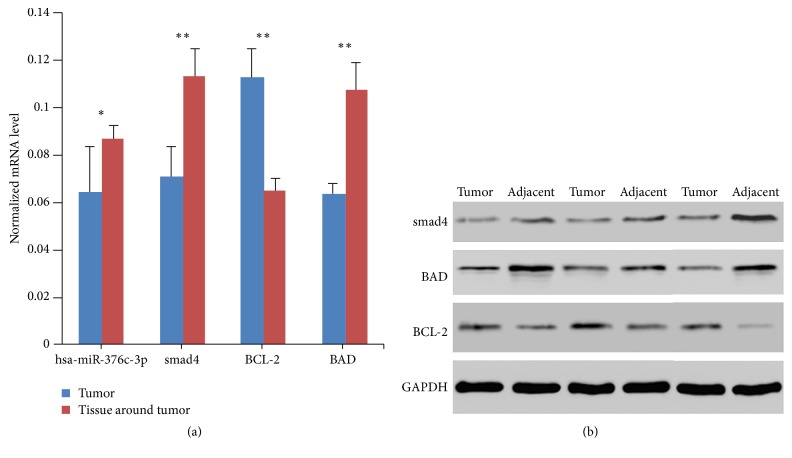
Expression change of hsa-miR-376c-3p, smad4, BAD, and BCL-2 in tumor tissue and tissue adjacent with tumor collected from gastric cancer patients was determined by real time PCR (a) and western blotting (b). The significance was calculated by paired *t*-test. ^*∗*^ *p* < 0.05; ^*∗∗*^ *p* < 0.01.

Finally, we collected three samples from three patients with gastric cancer. The real time PCR results indicated that the expression level of hsa-miR-376c-3p was significantly lower in tumor tissues compared to the tissues adjacent to tumors (Figure 6(a)). Furthermore, the mRNA expression of smad4 and BAD protein was significantly higher in adjacent tissues than in tumors, whereas the expression of BCL-2 was significantly lower in adjacent tissues than in tumors (Figure 6(a)). Similar results were obtained in all six patient samples in terms of the protein expression of BAD and BCL-2 (Figure 6(b)).

## 4. Discussion

The aberrant expression of miRNAs is involved in many diseases, including gastric cancer. In this study, we used hsa-miR-376c-3p inhibitor and overexpression construct to elucidate the role of hsa-miR-376c-3p in the gastric cancer cell apoptosis. Furthermore, we also indicated that overexpression of hsa-miR-376c-3p had an antigastric tumor effect* in vivo*.

In recent years, many studies have disclosed the dual role of miRNAs as oncogenes or tumor suppressor genes through activating multiple cellular signaling pathways, related with tumorigenesis, progression, and metastasis [17–20]. However, only limited number of studies showed specific miRNAs involved in human tumor formation* in vivo*. From this study, a previously unidentified miRNA hsa-miR-376c-3p was shown to be closely correlated with gastric cancer development and progression. More importantly, xenograft experiment indicated that gastric tumor growth was inhibited when the tumors were overexpressed in gastric cancer cells, suggesting that hsa-miR-376c-3p may serve as a tumor suppressor gene in gastric cancer.

Furthermore, our* in vitro* data showed that hsa-miR-376c-3p overexpression could lead to the promotion of apoptosis and G1 arrest in gastric cancer cells. This process was correlated with the increasing expression of BAD and decreasing expression of BCL-2. This suggested that hsa-miR-376c-3p induced apoptosis was mediated by BCL-2/BAD death pathway. BCL-2 is a crucial regulator of the mitochondrial dependent pathway of apoptosis. It has antiapoptotic function, preventing cytochrome c release from mitochondrion to cytosol [21]. The BCL-2-associated death promoter (BAD) protein is a proapoptotic member of the BCL-2 gene family which is involved in initiating apoptosis [22]. This raised the possibility that hsa-miR-376c-3p was a new target and provided a new strategy to treat malignant tumors, including gastric cancer. Further study will be focused on clarifying more specific mechanisms underlying the roles of hsa-miR-376c-3p in the progression and invasion of gastric cancer as well as to assess its function in clinical context.
